# Frequency of abnormal results in screening laboratory tests and associated interventions in emergency psychiatric services

**DOI:** 10.1002/pcn5.70093

**Published:** 2025-04-08

**Authors:** Shotaro Fujiwara, Takuto Ishida, Masafumi Mizuno

**Affiliations:** ^1^ Department of Psychiatry Tokyo Metropolitan Matsuzawa Hospital Tokyo Japan; ^2^ Department of Internal Medicine Tokyo Metropolitan Matsuzawa Hospital Tokyo Japan

**Keywords:** emergency psychiatric service, psychomotor agitation, suicide attempt, routine diagnostic test, medical clearance

## Abstract

**Aim:**

Screening laboratory tests for patients with acute psychiatric symptoms are discouraged in general emergency services but are recommended by emergency psychiatric guidelines due to the high prevalence of abnormal findings in severe psychiatric patients. The present study aimed to evaluate the utility of screening laboratory tests in a real‐world, emergency psychiatric setting through focusing on how often abnormal values lead to medical interventions.

**Methods:**

The electronic medical records were reviewed for adult patients who were involuntarily admitted to Tokyo Metropolitan Matsuzawa Hospital for an imminent risk of self‐harm or harm to others between October 2019 and September 2022. The number of patients, abnormal screening laboratory findings, and medical interventions were examined.

**Results:**

Of the 600 patients identified in the review, 595 had abnormal laboratory findings, but only 97 (16.3%) underwent medical interventions related to these results. Frequently observed abnormal findings, such as elevated creatine kinase and an elevated white blood cell count, were often attributed to the patient's agitation or were considered clinically nonsignificant. Notably, one‐third of the interventions prompted by laboratory findings resulted only in additional testing. More than half of the treatments were either of questionable necessity or nonurgent. Medical interventions other than additional tests were more likely to be prompted by the patient's medical history and presentation.

**Conclusion:**

In the emergency psychiatric setting, abnormal laboratory findings are common, but clinical decisions largely rely on the patient's medical history and presentation, with few requiring immediate interventions. A history‐driven approach may enhance the clinical value of laboratory tests.

## Graphical abstract

Severe psychiatric patients often have abnormal laboratory test findings. Most abnormalities are attributed to agitation or are clinically insignificant, and interventions are limited. Medical intervention was often promoted by the patient's medical history.

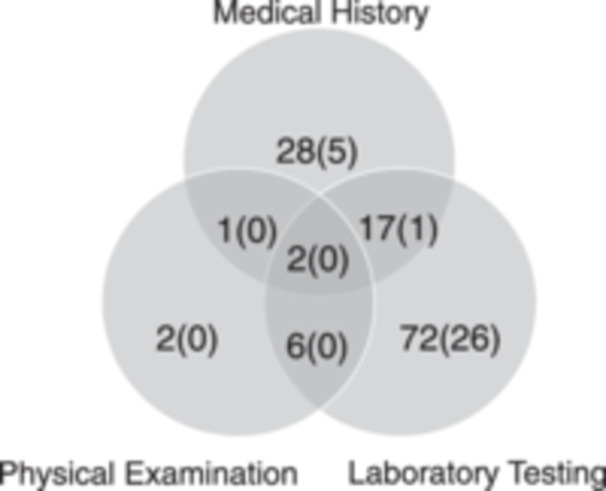

## INTRODUCTION

Screening laboratory tests for acute psychiatric symptoms are discouraged in general emergency medicine. Previous studies demonstrated that screening laboratory tests rarely changed the patient's disposition or led to medical interventions.^1^, ^2^, ^3^, ^4^, ^5^ Therefore, the general emergency guidelines recommend that laboratory tests for patients with acute psychiatric symptoms should not be routinely administered but rather be ordered on the basis of the patient's medical history, previous psychiatric diagnoses, and physical examination findings.^6^ Furthermore, screening laboratory tests can have several negative consequences, including the omission of medical interviews or physical examinations,^7^ the failure to review or act upon test results, and unnecessary medical tests and treatments.^8^ There is currently a movement known as the “choosing wisely campaign,” which advocates improving the quality of healthcare by reducing the wastage of medical resources, minimizing the risks associated with unnecessary medical interventions, and promoting more appropriate testing.^9^, ^10^, ^11^, ^12^

However, it remains unclear whether these recommendations regarding screening laboratory tests for patients with psychiatric symptoms also apply to patients with severe psychiatric symptoms in the emergency psychiatry setting. Previous studies have excluded patients with severe psychiatric symptoms, such as those who had attempted suicide^3^ or exhibited violent agitation.^4^ In the real‐world setting, patients with severe psychiatric symptoms often refuse medical interviews and physical examinations, and psychiatrists sometimes omit these procedures owing to the difficulty of performing them or for their own safety.^13^, ^14^ Because patients with severe psychiatric symptoms sometimes have physical comorbidities, some clinical protocols recommend performing laboratory tests.^15^, ^16^, ^17^, ^18^, ^19^ However, laboratory tests for aggressive, uncooperative, or agitated patients should be minimized owing to an increased risk of needlestick injuries.^20^, ^21^ In fact, some institutions have the policy of not routinely performing laboratory tests for agitated patients.^22^

The present study aimed to assess the value of screening laboratory tests for patients who were involuntarily transferred and admitted because they posed an imminent risk of harming themselves or others. Our hypothesis is that screening laboratory tests have as little impact on medical interventions in the psychiatric emergency setting as they do in the general emergency setting.

## METHODS

### Study design and setting

The present, monocentric, retrospective cohort study was conducted at Tokyo Metropolitan Matsuzawa Hospital, a psychiatric center providing involuntary and emergency psychiatric evaluations in Tokyo, Japan. Patients posing an imminent risk of harm to themselves or others are involuntarily transferred to this hospital by several police officers. During the off‐hours (5 p.m.–8 a.m.), a licensed psychiatrist makes the decision on involuntary admission. Screening laboratory tests are routinely conducted in the emergency psychiatric room at admission. After the decision on involuntary hospitalization is approved by two additional licensed psychiatrists during normal operating hours on the following day, a patient without a physical complaint is transferred to a psychiatric hospital, which has limited laboratory testing facilities. Involuntary hospitalization is conducted under the Act on Mental Health and Welfare for Persons with Mental Disorders or Disabilities. There are about 200 cases of involuntary emergency admission yearly at the study center.

### Patients

The present study included patients aged 18 years or older who were involuntarily hospitalized at Tokyo Metropolitan Matsuzawa Hospital between October 1, 2019, and September 30, 2022, during the off‐hours because they posed an imminent risk of harm to themselves or to others. The exclusion criteria were admission after undergoing a physical evaluation or treatment at another general hospital; refusal to participate in this study; and no laboratory tests in the emergency psychiatric room.

### Outcome measures

The primary outcome measure was the proportion of patients with a medical intervention related to abnormal laboratory findings, which was calculated by examining the number of each screening laboratory test item, the number of abnormal findings per item, and the number of medical interventions related to these findings. “Medical intervention” was defined as an unscheduled, additional test or physical treatment regardless of its necessity or importance. Intervention for injuries (e.g., fractures from jumping or wounds from wrist‐cutting) was not considered as a medical intervention but was counted separately because these conditions do not require screening laboratory tests for their diagnosis. An “abnormal laboratory finding” was defined as a laboratory test result falling outside the range considered normal at the study center.

The secondary outcome was the basis for the medical interventions, which was categorized into medical history, physical examination findings, screening laboratory test findings, or a combination thereof. The details of the treatments performed solely on the basis of laboratory tests were documented.

### Data collection and analysis

The patients' electronic medical records were retrospectively reviewed. The charts were first analyzed by a single observer, whose analysis was then confirmed by an independent emergency physician. In the event of a disagreement, a discussion was held by the reviewers until a consensus was reached. Table 1 lists the components of the screening laboratory tests.

**Table 1:** Screening laboratory test categories and items.

Hematology	WBC, Hb, Plt, D‐dimer
Biochemistry	Total protein, albumin, CK, sodium, chloride, potassium, calcium, ionized calcium, magnesium, BUN, creatinine, AST, ALT, LDH, ALP, γGTP, total bilirubin, amylase, glucose, HbA1c (NGSP), TSH, free T4
Infectious diseases	RPR, TPLA, HBs Ag, HCV index, HIV Ag/Ab index

Abbreviations: ALP, alkaline phosphatase; ALT, alanine aminotransferase; AST, aspartate aminotransferase; BUN, blood urea nitrogen; CK, creatine kinase; free T4, free thyroxine; Hb, hemoglobin; HbA1c (NGSP), hemoglobin A1c (national glycohemoglobin standardization program); HBs Ag, hepatitis B surface antigen; HCV index, hepatitis C virus index; HIV Ag/Ab Index, human immunodeficiency virus antigen/antibody index; LDH, lactate dehydrogenase; Plt, platelets; RPR, rapid plasma reagin; TPLA, treponema pallidum particle agglutination; TSH, thyroid‐stimulating hormone; WBC, white blood cells; yGTP, gamma‐glutamyl transferase.

The attending physicians' evaluations of common laboratory abnormalities were reviewed, and the severity of common laboratory abnormalities with medical interventions was assessed.

The following clinical information was collected from the patients' profiles: age, sex, presence of a family member or acquaintance at the admission interview, psychiatric history, vital signs (respiratory rate [breaths/min], systolic and diastolic blood pressure [mm Hg], heart rate [beats/min], body temperature [°C], oxygen saturation [%]), and presence of pharmacological sedation and its administration route. Numerical values were expressed as the average ± standard deviation (SD). To determine whether any abnormal vital signs were associated with a medical intervention, patients with vital signs indicative of the need for further diagnostic tests were counted.^15^

## RESULTS

### Patient profile

In 3 years, 666 patients were involuntarily hospitalized. Of these, 64 were excluded because they were admitted after receiving a medical intervention at another general hospital; one was excluded because laboratory tests were omitted due to the needlestick risk during an agitated state; and one was excluded owing to the lack of medical records. Thus, 600 patients (316 females [52.7%]) were finally included. The mean age was 45.0 ± 15.8 years (1 SD) (*n* = 595; age was unknown for five patients). Apart from 16 patients who received an intervention for an injury, 128 patients (21.3%) received some form of medical intervention. Police officers and the patients themselves were the only sources of information in 307 (51.2%) cases, while family members or acquaintances were also informants in the remaining 293 (48.8%) cases. At the time of the admission interview, 446 (74.3%) patients had a known psychiatric history. All the patients had a Global Assessment of Functioning score <20. None of the patients returned to the study center with physical symptoms following their transfer to another psychiatric hospital.

### Screening laboratory tests

Table 2 summarizes the characteristics of each laboratory test and the number of patients with a medical intervention. Of the 600 patients, 595 (99.2%) had abnormal laboratory findings, but only 97 (16.3%) received a medical intervention related to these results. Common abnormalities were increased lactate dehydrogenase (LDH) (*n* = 378), white blood cells (WBC) (*n* = 348), and creatine kinase (CK) (*n* = 336). Abnormal laboratory test results that commonly led to a medical intervention (either a treatment or additional testing) were elevated CK (*n* = 33), abnormal glucose metabolism (*n* = 19 for blood glucose level; *n* = 18 for HbA1c), and electrolyte imbalance (*n* = 13 for sodium; *n* = 14 for potassium).

**Table 2:** Number of each laboratory test, abnormal values, and medical interventions (treatments and additional tests).

Laboratory test	Normal range	Number of tests	Number of abnormal findings				
				Action taken			
				[% of abnormal findings related to intervention]		Additional tests	Medical treatments
Hematology							
WBC	3.30–8.60 × 10^3^/mm^3^	600	348	4	[1.1]	1	3
Hb	11.6–14.8 gm/dL female	600	139	7	[5.0]	2	5
	13.7–16.8 gm/dL male						
Plt	158–348 × 10^3^/mm^3^	600	113	0	[0.0]	0	0
D‐dimer	<1.0 μg/mL	600	164	7	[4.3]	3	4
Biochemistry							
Total protein	6.6–8.1 g/dL	599	106	0	[0.0]	0	0
Albumin	4.10–5.10 g/dL	600	159	0	[0.0]	0	0
CK	41–153 units/L female	600	336	33	[9.8]	10	23
	59–248 units/L male						
Sodium	138–145 mEq/L	600	151	13	[8.6]	6	7
Chloride	101–108 mEq/L	600	125	1	[0.8]	1	0
Potassium	3.6–4.8 mEq/L	600	169	14	[8.3]	6	8
Calcium	8.8–10.1 mg/dL	596	100	0	[0.0]	0	0
Ionized calcium	8.8–10.1 mg/dL	596	88	1	[1.1]	0	1
Magnesium	1.8–2.4 mg/dL	600	75	1	[1.3]	0	1
BUN	8.0–20.0 mg/dL	600	184	13	[7.1]	3	10
Creatinine	0.46–0.79 mg/dL female	600	107	8	[7.5]	0	8
	0.65–1.07 mg/dL male						
AST	13–30 units/L	600	228	0	[0.0]	0	0
ALT	7–23 units/L female	600	178	0	[0.0]	0	0
	10–42 units/L male						
LDH	124–222 units/L	600	378	0	[0.0]	0	0
ALP	38–113 units/L	501	36	0	[0.0]	0	0
γGTP	9–32 units/L female	599	143	0	[0.0]	0	0
	13–64 units/L male						
Total bilirubin	0.40–1.50 mg/dL	598	97	0	[0.0]	0	0
Amylase	44–132 units/L	597	137	1	[0.7]	0	1
Glucose	73–109 mg/dL	600	240	19	[7.9]	3	16
HbA1c (NGSP)	4.9%–6.0%	599	129	18	[14.0]	1	17
TSH	0.350–4.940 μ units/mL	599	46	2	[4.3]	0	2
Free T4	0.70–1.48 ng/dL	599	27	1	[3.7]	0	1
Infectious diseases							
RPR	0.0–0.9 RU	599	7	1	[14.3]	0	1
TPLA	0.0–1.0 COI	599	9	1	[11.1]	0	1
HBs Ag	<0.05 units/mL	599	5	0	[0.0]	0	0
HCV index	<1.00 S/CO	599	13	0	[0.0]	0	0
HIV Ag/Ab index	<1.00 S/CO	589	0	0	[n.a.]	0	0
Total	Abnormal value	600	595	97	[16.3]	27	70

*Note*: Ionized calcium is calculated with Payne's formula as “(Ca (mg/dL) + (4 ‐ Alb (g/dL)).”

Abbreviations: ALP, alkaline phosphatase; ALT, alanine aminotransferase; AST, aspartate aminotransferase; BUN, blood urea nitrogen; CK, creatine kinase; free T4, free thyroxine; Hb, hemoglobin; HbA1c (NGSP), hemoglobin A1c (national glycohemoglobin standardization program); HBs Ag, hepatitis B surface antigen; HCV index, hepatitis C virus index; HIV Ag/Ab Index, human immunodeficiency virus antigen/antibody index; LDH, lactate dehydrogenase; Plt, platelets; n.a., not applicable; RPR, rapid plasma reagin; TPLA, treponema pallidum particle agglutination; TSH, thyroid‐stimulating hormone; WBC, white blood cells; yGTP, gamma‐glutamyl transferase.

#### WBC and LDH

Of the 347 cases of elevated WBC, the medical history and physical findings ruled out the need for a medical intervention in 166 cases, while 65 cases were attributed to the patients' agitation, 13 to suspected infection, and six to an injury. The remaining 97 cases were unassessed. One case of decreased WBC count was determined to be a symptom of liver cirrhosis. Consequently, only four patients (1.1%) underwent a medical intervention. There was no assessment of abnormal LDH.

#### Creatine kinase

Of the 322 cases of elevated CK, the medical history and physical findings ruled out the need for medical intervention in 117 cases, while 97 cases were attributed to the patients' agitation, two to hemolysis, one to being in a supine position for a prolonged period, and one to hyponatremia. The remaining 104 cases were unassessed. Of these, 14 patients had a CK level exceeding 5000 U/L, which is the diagnostic and treatment threshold for rhabdomyolysis.^23^, ^24^ All cases of CK > 5000 U/L were attributed to the patients' agitation except for one case, which was induced by hyponatremia. Twelve patients received fluid therapy, and two received only the repeated laboratory test on the following day.

#### Abnormal blood glucose metabolism

Seventeen patients met the diagnostic criteria for diabetes (both random blood glucose ≥200 mg/dL and HbA1c ≥ 6.5%). Half of these patients received nonemergency, follow‐up blood glucose monitoring or insulin therapy after admission while the remaining half had their laboratory test data transferred to a psychiatric hospital. Ten patients had hypoglycemia with a random blood glucose level of 70 mg/dL or less. Three of these patients received nonemergency glucose after admission, two received hypoglycemia treatment, and the remaining five, who had normal hospital meal intake and no medical intervention, were transferred to another psychiatric hospital. No patient had an abnormal blood glucose level associated with psychiatric symptoms or received emergency treatment; the attending physician assessed the patients on the basis of psychiatric history, behavior, and the episode timeline.

#### Electrolyte abnormalities

Electrolyte abnormalities were classified as moderate or severe in accordance with previous studies.^25^, ^26^ Moderate and severe hyponatremia were defined as Na 125–129 mEq/L and Na < 125 mEq/L, respectively; moderate and severe hypernatremia were defined as Na 150–169 mEq/L and Na ≥ 170 mEq/L, respectively; moderate and severe hypokalemia were defined as K 2.5–3.0 mEq/L and K < 2.5 mEq/L, respectively; and moderate and severe hyperkalemia were defined as K 6.0–7.0 mEq/L and K > 7.0 mEq/L, respectively.

Moderate hyponatremia, hypernatremia, hypokalemia, and hyperkalemia were observed in four, three, 21, and two patients, respectively. Five patients had severe hyponatremia; of these, four had the condition due to water intoxication, and one due to poor oral intake. Two of the patients received fluid therapy while three underwent only additional laboratory tests. None of the patients had a sodium level exceeding 160 mEq/L, the threshold for urgent treatment according to the Japanese Society of Internal Medicine.^27^ The attending psychiatrist assessed all electrolyte abnormalities as a consequence rather than a cause of psychiatric symptoms on the basis of information from informants. Two patients had severe hypokalemia caused by inadequate intake and received fluid therapy. Severe hyperkalemia was observed in one patient but was attributed to hemolysis and was only followed up with a laboratory test on the following morning. Importantly, 27 of 600 laboratory tests found hemolysis.

### Basis of medical interventions

**Figure 1:**
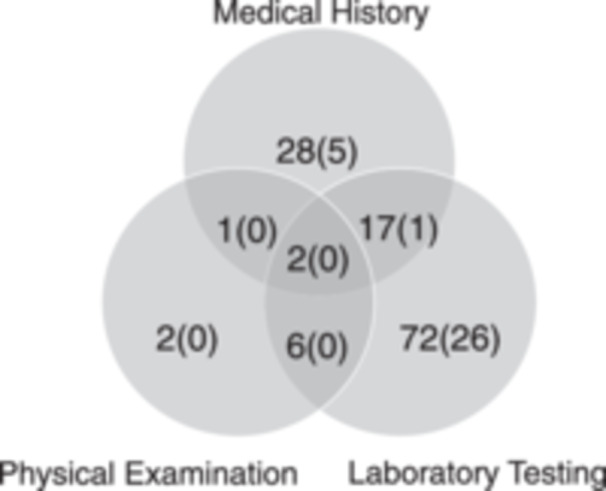
Medical interventions and their basis. Each number in parentheses refers to the number of additional tests only.

Of the 128 medical interventions, 56 were based on clinical information (28 on the medical history, two on physical examination findings, and 26 on a combination of the medical history, physical examination, and laboratory test findings), while 72 were based solely on laboratory test findings (Figure 1). Additional tests were conducted for 36.1% (26/72) of the medical interventions prompted by laboratory test findings and for 10.7% (6/56) of those prompted by clinical findings.

Among the 46 patients who received a medical intervention other than additional tests based on laboratory results alone, 20 received an intervention of unclear necessity, including fluid therapy despite CK < 5000 U/L (as low as 250 U/L in one patient) (*n* = 14), fluid therapy for elevated blood urea nitrogen (BUN) alone (*n* = 4), and an intravenous infusion of glucose for mild hypoglycemia despite being capable of oral intake (*n* = 2). Additionally, 12 patients received a nonurgent intervention or judgment, such as diabetes management without acute complications (*n* = 4), oral iron for anemia (*n* = 4), oral potassium supplementation for mild hypokalemia (*n* = 2), syphilis (non‐neurosyphilis) treatment postponed to daytime (*n* = 1), and a new diagnosis of hyperthyroidism without urgent treatment (*n* = 1). In contrast, 14 patients received an appropriate intervention, including fluid therapy for CK > 5000 U/L (*n* = 11), fluid therapy for hypernatremia (Na 154 mEq/L; *n* = 1), water restriction for suspected water intoxication (Na 112 mEq/L; *n* = 1), and treatment for hyperkalemia (K 6.5 mEq/L; *n* = 1).

### Vital signs and psychiatric de‐escalation

All vital signs were recorded in 363 patients (60.5%) in the emergency room. The reason for not recording the vital signs was noted as “violent or uncooperative behavior.” Table 3 presents the average vital sign values. In total, 181 patients had vital signs outside the threshold for further testing as described in previous studies^15^ (Table 3). Of these 181 patients, only 45 (24.9%) received a medical intervention.

**Table 3:** Number and proportion of patients with abnormal vital signs.

Vital signs	Number	Average (±1 SD)	Abnormal range	Abnormal number	Medical intervention [% of patients with abnormal values]	
Respiratory rate (/min)	392	18.2 ± 3.4	Lower than 8 or higher than 22	0 37	0 6	[n.a.] [16.2]
Systolic blood pressure (mmHg)	466	132.3 ± 24.2	Lower than 100 or higher than 180	31 17	5 2	[16.1] [11.8]
Diastolic blood pressure (mmHg)	466	86.2 ± 18.1	Higher than 110	39	4	[10.3]
Pulse rate (/min)	497	92.0 ± 19.7	Lower than 50 or higher than 110	2 80	0 24	[0.00] [30.0]
Temperature (°C)	466	37.0 ± 3.9	Higher than 38	11	6	[54.5]
SpO_2_ (room air) (%)	509	96.9 ± 5.3	More than 95	28	11	[39.3]
Total	578		Abnormal value	181	45	[24.9]

The table shows the number of vital signs recorded, their average value, and the number of abnormal values and their range. An abnormal vital sign was defined by a value outside the threshold for further diagnostic testing in accordance with previous studies.^15^ Of patients with each abnormal vital sign, the number and percentage of patients with abnormal findings who are receiving medical intervention was calculated.

Abbreviation: n.a.: not applicable.

In terms of psychiatric de‐escalation, 197 patients (32.8%) were de‐escalated using nonpharmacological methods, while 403 (67.2%) were pharmacologically sedated on admission. Most of the latter (385/403, 95.5%) received an intravenous sedative while the rest received an oral sedative.

## DISCUSSION

The present, retrospective study found that despite nearly all patients with severe psychiatric symptoms having abnormal laboratory results, only 97 (16.3%) received a medical intervention. Most patients with abnormal findings who received no intervention were managed on the basis of their medical history and clinical assessment rather than laboratory findings, emphasizing the greater role of the medical history in clinical decision‐making. Furthermore, one‐third of these medical interventions prompted by laboratory tests were only additional tests. Among the 46 patients who received treatment solely on the basis of screening laboratory results, 32 received nonurgent or excessive treatment while only 14 received clinically beneficial care. Consequently, the abnormal laboratory findings per se had little impact on the patients' medical management. To the best of our knowledge, this study is the first to assess the value of screening laboratory tests in real‐world emergency psychiatric practice for patients with an imminent risk of harm to themselves or to others. The findings of the present study suggested the need to carefully re‐evaluate the utility of screening laboratory tests in the emergency psychiatric setting from several perspectives.

### Discontinuity between abnormal screening laboratory findings and medical interventions

Although abnormal laboratory findings are common in the emergency psychiatric setting, they only occasionally led to medical intervention. Previous studies in the emergency psychiatric setting demonstrated the importance of laboratory testing^28^, ^29^, ^30^ because patients with severe psychiatric symptoms frequently have abnormal test results, such as elevated CK, WBC, BUN, and creatinine and electrolyte abnormalities. Although the present findings aligned with those previous studies, most abnormal values, such as elevated CK and WBC, were found to be unproblematic or were attributed to the patients' agitation. This disconnection between abnormal test results and medical interventions highlights the primacy of medical history in the decision‐making process, as laboratory abnormalities alone rarely justify intervention.

### Negative consequences of screening laboratory tests

While screening laboratory tests are intended to aid clinical decision‐making, they can also have unintended negative consequences. Previous studies have shown that too many tests and too many abnormal results can habituate healthcare workers, leading to the inadvertent omission or misinterpretation of clinically significant results^31^ and unnecessary medical procedures.^8^ In the present study, common laboratory abnormalities were often attributed to the patients' agitation or were not assessed; however, this attitude might have been influenced by a cognitive bias due to repeated abnormal test results. Additionally, blood collection in agitated psychiatric patients poses significant risks. Because one‐quarter to one‐half of needlestick injuries are attributed to the patient's uncooperativeness, movements, or aggression,^20^, ^21^ collecting blood from patients with severe psychiatric symptoms increases the risk of needlestick injuries. Furthermore, appropriately collecting blood from severe psychiatric patients is also challenging owing to these patients' susceptibility to hemolysis, which in turn often leads to unnecessary, additional laboratory testing.^32^, ^33^ In the present study, the proportion of hemolytic samples was 4.5%, which is more than double the general benchmark of 2%.^34^, ^35^ The high hemolysis rate might have been caused by excessive negative pressure applied to the syringe while collecting blood from agitated patients in an effort to expedite the procedure.

### Optimizing laboratory testing: Moving from routine screening tests to selective and appropriate testing

Optimized laboratory testing can occasionally guide critical interventions in a small subset of patients with severe psychiatric symptoms who may be unable to communicate effectively. Unlike the complete uselessness of screening in general emergency medicine,^1^, ^2^, ^3^, ^4^, ^5^ 14 patients in the present study received clinically beneficial treatment based solely on laboratory findings. To enhance the utility of laboratory tests while minimizing unnecessary testing, clinicians must determine whether immediate testing is necessary or can be safely deferred. Further research is needed to establish evidence‐based strategies for optimization.

The medical history remains the most critical tool for guiding medical interventions and optimizing laboratory tests in psychiatric emergencies. Both general and psychiatric emergency medicine emphasize the importance of medical history, a physical examination, and assessment of the vital signs.^1^, ^2^, ^3^, ^4^, ^5^, ^6^, ^15^, ^16^, ^17^, ^18^, ^19^ In the present study, most patients with abnormal test results who did not receive a medical intervention were evaluated on the basis of their medical history and presentation. In addition, 4% (24/600) of the patients received a medical intervention based on their medical history alone (Figure 1), whereas only 0.3% (2/600) of patients received a medical intervention based solely on their physical examination findings. In addition, 39.5% of the patients were unwilling or unable to cooperate with their all‐vital‐sign measurements, and only 24.9% (45/181) of the patients with abnormal vital signs received an additional medical intervention. These findings highlight the difficulty of evaluating the vital signs and performing physical examinations in involuntary psychiatric assessments, where misinterpretation is common. Even in psychiatric emergencies where mental symptoms dominate, comprehensive history‐taking, including physical information, is more important than an overreliance on routine screening tests.

### Limitations

The present study has several limitations. First, it was monocentric and retrospective and therefore prone to biases. Second, the appropriateness of the medical interventions, treatments, and psychiatrists' assessments of the laboratory test results were unable to be fully evaluated using medical records alone; thus, the need for medical interventions may have been over‐ or underestimated. Third, this study was based on a review of medical records with a limited number of patients; therefore, a larger sample study might have revealed negative outcomes resulting from overlooking latent physical illnesses. Fourth, the data review process did not involve independent dual assessment, and inter‐rater reliability was unable to be calculated, thereby possibly introducing observer bias. Fifth, only the absolute level of electrolytes was measured although the speed of the changes in the electrolyte values, clinical symptoms, and electrocardiogram findings are more relevant clinically. Sixth, the association between the laboratory screening tests and long‐term outcomes was unable to be examined; thus, the utility of the screening laboratory tests may have been underestimated. Seventh, the utility of the laboratory test findings in ruling out physical conditions was not considered; normal findings not affecting the patients' management might have helped clinicians to rule out certain physical conditions. Eighth, the number of patients without a physical examination was not determined, thus possibly leading to an underestimation of the utility of physical examinations. Ninth, this study did not include a control group; therefore, no statistical evaluation of the effectiveness of the tests was able to be performed. Finally, the vital signs were not assessed in all the patients, possibly leading to an underestimation of their significance despite this being reflective of the real‐world clinical practice in the emergency psychiatric setting.

## CONCLUSION

Screening laboratory tests in the emergency psychiatric setting often lead to redundant findings of abnormalities, which in turn can prompt further, unnecessary testing or interventions. Considering the risks and staffing requirements for the laboratory testing of patients with severe psychiatric symptoms, a more selective approach is necessary. To minimize unnecessary testing and ensure that necessary tests are conducted safely and effectively, clinicians should obtain a detailed medical history to assess the need for immediate testing, and implement psychiatric de‐escalation strategies to optimize the conditions for laboratory testing. Further research is necessary to develop evidence‐based criteria for identifying patients who truly require urgent laboratory evaluation.

## AUTHOR CONTRIBUTIONS

All the authors have contributed to the study's conception and design. Material preparation and data collection and analysis were performed by Shotaro Fujiwara and Takuto Ishida. The first draft of the manuscript was written by Shotaro Fujiwara and all authors have commented on previous versions of the manuscript. All authors have read and approved the final manuscript.

## CONFLICT OF INTEREST STATEMENT

Shotaro Fujiwara and Masafumi Mizuno have no conflicts of interest. Takuto Ishida received a manuscript fee from Sumitomo Pharma within the past 3 years. The authors have no relevant employment, financial, or nonfinancial interests to disclose.

## ETHICS APPROVAL STATEMENT

This study was approved by the institutional review board of Tokyo Metropolitan Matsuzawa Hospital (No. 2022‐17), was conducted according to the principles of the Declaration of Helsinki, and was exempted from informed consent due to the use of de‐identified data acquired during routine clinical care.

## PATIENT CONSENT STATEMENT

As this was a retrospective study, an opt‐out approach was adopted, with research objectives made public, allowing potential participants to refuse participation.

## CLINICAL TRIAL REGISTRATION

This study was a retrospective analysis. Therefore, the criteria for clinical trial registration do not apply.

## Data Availability

Data availability statement: The data that support the findings of this study are not openly available due to reasons of sensitivity but are available from the corresponding author upon reasonable request. The data are in controlled access data storage at Tokyo Metropolitan Matsuzawa Hospital.
